# PERSONS LIVING WITH DEMENTIA AND MULTIPLE CHRONIC CONDITIONS IDENTIFYING HEALTH PRIORITIES WITH CARE PARTNERS

**DOI:** 10.1093/geroni/igad104.1374

**Published:** 2023-12-21

**Authors:** Thi Vu, Emily Mroz, Kizzy Hernandez-Bigos, Denise Chow, Rafael Samper-Ternent, Kalisha Bonds Johnson, Mary Tinetti, Joan Monin

**Affiliations:** Yale University School of Public Health, New Haven, Connecticut, United States; Yale School of Medicine, New Haven, Connecticut, United States; Yale University School of Medicine, New Haven, Connecticut, United States; Yale University School of Public Health, New Haven, Connecticut, United States; UTHealth Houston, Houston, Texas, United States; Emory University, Atlanta, Georgia, United States; Yale School of Medicine, New Haven, Connecticut, United States; Yale School of Public Health, New Haven, Connecticut, United States

## Abstract

Many older persons living with dementia (PLWD) also live with multiple chronic conditions (MCCs). Care partners of PLWD will increasingly need to make care decisions for PLWD, but the health care priorities of PLWD with MCCs may vary. The Patient Priorities Care Health Priorities Identification Process is an evidenced-based tool to support older adults with MCCs to identify and communicate their health priorities. In this pilot study, we adapted this tool to engage care partners of PLWD as active listeners while the PLWD discussed their healthcare goals and priorities. We recruited dyads consisting of a care partner and a care recipient in the early stages of dementia. We assessed feasibility and acceptability of going through the process together through open-ended questions. Two coders used thematic analysis to examine their responses. Five dyads (N=10) participated in the pilot study. The PLWD ranged in age from 70-83 years and had a range of three to eight chronic conditions. Care partners ranged from 39-78 years of age. The PLWD had no issues completing the process with their care partners present and enjoyed having their care partner present. Care partners valued engaging as active listeners and had a better understanding of the PLWD’s health care priorities. Engagement in this process also helped them recognize that the PLWD is proactive in their own health goals. Involving care partners in a healthcare priorities identification process is a valuable, feasible, and acceptable way to prepare care partners to be care proxies.

